# Seropositivity and Coinfection of Hepatitis B and C among Patients Seeking Hospital Care in Islamabad, Pakistan

**DOI:** 10.1155/2014/516859

**Published:** 2014-06-24

**Authors:** Jafar Khan, Mehwish Shafiq, Sameera Mushtaq, Sultan Ayaz, Riaz Ullah, Naser M. AbdEI-Salam, H. Fouad, Mohammad Abdul Wasim

**Affiliations:** ^1^Department of Microbiology, Kohat University of Science and Technology, Kohat 26000, Khyber Pakhtunkhwa, Pakistan; ^2^Department of Zoology, Kohat University of Science and Technology, Kohat 26000, Pakistan; ^3^Department of Chemistry, Government College Ara Khel, FR Kohat 26000, Pakistan; ^4^Riyadh Community College, King Saud University, Riyadh 11437, Saudi Arabia; ^5^Department of Chemistry, Sarhad University of Science and Information Technology, Peshawar 25000, Pakistan

## Abstract

The undertaken study was conducted to find out the seroprevalence and coinfection of HBV and HCV infection among patients seeking hospital care. A total of 845 samples were received at tertiary care hospital of Islamabad and were screened for hepatitis B and C. The ELISA was used to detect antigen for HBV and antibodies for HCV in patient serum. Among 845 collected samples, 255 (30.1%) were seropositive for HBV and HCV. Out of 255 seropositive samples, 45 (5.3%) were positive for HBsAg while 199 (23.5%) were positive for anti-HCV. Among 255, 11 (1.3%) were seropositive for both HBsAg and anti-HCV (coinfection). Among the seropositive male, HBV was more prevalent (23.8%) while female patients had a high incidence of HCV (52.2%). Among the age group variable, HBV, HCV, and coinfection were found to be more common in the age groups of 21–30 (29%) and 30–40 (24%) years. The seropositivity for HBsAg was higher in unmarried individuals (31.2%) while anti-HCV was more prevalent in married individuals (84%). The present study provides the preliminary information about high HCV and HBV prevalence. Findings from the current study will be helpful for the better management and control of viral hepatitis among patients seeking hospital care.

## 1. Introduction

Viral hepatitis is a major health problem in all parts of world. HBV and HCV are hepatotropic viruses leading to significant morbidity and mortality worldwide [1, 2]. HBV is a member of Hepadnaviridae family harboring a DNA genome while HCV is RNA virus that belongs to Flaviviridae family [3].

Hepatitis B and C are transmitted parentally mainly as a result of blood contact, including injury with contaminated instruments and sharing of needles, or by sexual contact and also through parental transmission from mother to child [4]. Hepatitis B and C infections can lead to an acute or silent course of liver disease progressing from liver impairment to liver failure, cirrhosis of liver, and hepatocellular carcinoma [1, 2].

The global prevalence of HCV is about 2.8%; while more than 185 million people are infected with HCV alone, HBV prevalence is variable around the globe; however, among the 2 billion people infected with HBV, about 360 million people are chronic carriers around the world [2, 5]. South and Southeast Asian countries have estimated prevalence rate from 1.5 to 3.5% [2]. In Pakistan the seroprevalence of hepatitis B surface antigen (HBsAg) and HCV antibodies is about 2.5% and 4.8%, respectively, with an overall infection rate of 7.6% in the general population [6].

HBV and HCV may appear as coinfection due to the same mode of transmission [4, 7]. Several studies documented that HBV and HCV coinfection accelerates liver disease progression and increases the risk of hepatocellular carcinoma [7–9], and the patients need high dose of interferon treatment [10]. Although a growing body of the literature is available on the prevalence of HCV and HBV [6, 11, 12], limited data is documented on the coinfection of HBV and HCV from Islamabad.

The present study reported the seroprevalence and coinfection of HBV and HCV among patients seeking hospital care in Islamabad. Findings from this study may be helpful to formulate strategy for the prevention of HBV and HCV coinfection.

## 2. Materials and Methods

### 2.1. Study Area

The study was conducted from 1st July to 31st August, 2011, at Pakistan Institute of Medical Sciences (PIMS), Islamabad. All the patient seeking hospital care were enrolled in the study.

### 2.2. Sample Collection

During this period a total of 845 blood samples from patients seeking hospital care suspected for viral hepatitis were collected in sterilized vacutainer, dully labeled with sex, age, areas, and date of collection, and kept in refrigerator at −20°C for further process.

### 2.3. Sample Screening

A total of 845 blood samples were screened for detection of HBV and HCV using ICT (immunochromatography test). The screen samples were further subjected to ELISA for reconfirmation of the test.

### 2.4. Enzyme Linked Immunosorbent Assay (ELISA)

For detection of HBsAg and HCV antibodies two types of ELISA kits were used. HbsAg ELISA kit is an enzyme-linked immunosorbent assay (ELISA) for qualitative detection of HBsAg in human serum or plasma. For detection of HBsAg with ELISA kit, sandwich ELISA method was used. HCV ELISA kit is an enzyme-linked immunosorbent assay for qualitative detection of antibodies to hepatitis C virus in human serum or plasma. For HCV antibody detection indirect ELISA was employed. The Biokit ELISA system (BEST 2000) was used for running ELISA.

### 2.5. Data Analysis

The data was analyzed with Window 7, Microsoft Excel 2007 (Microsoft, USA).

## 3. Results and Discussion

Of total 845 collected samples, 255 (30.1%) samples were positive for hepatitis B and C. Out of 255 samples, 45 (5.3%) were positive for HBsAg while 199 (23.5%) were positive for anti-HCV. Among 255, 11 (1.3%) were seropositive for both HBsAg and anti-HCV (coinfection) (Table 1). Among the seropositive male (*n* = 122, 47.8%), HBV was more prevalent (*n* = 29, 23.8%) while female patients (*n* = 112, 84.2%) had a high frequency of HCV (*n* = 133, 52.2%). The coinfection rate was higher in male individuals (*n* = 06, 4.9%) as compared to female patients (*n* = 05, 3.8%) (Table 2). Among the age group variable HBV, HCV, and coinfection were found to be more frequent in the age groups of 21–30 (*n* = 74, 29%) and 30–40 (*n* = 63, 24%) years while age groups of 1–9 (*n* = 04, 02%) and 70–80 (*n* = 04, 02%) years are less infected by HBV and HCV (Table 3). The seropositivity for HBsAg was higher in unmarried individuals (*n* = 25, 31.2%) while anti-HCV was more prevalent in married individuals (*n* = 147, 84%) (Table 4). Moreover, samples collected from General (*n* = 87, 34.1%) and Gastroenterology 54 (21.1) ward showed increased HCV and HBV infection rates. The samples collected from Pulmonology ward showed less seropositivity for HBV and HCV (*n* = 03, 1.1%) markers (Table 5). Viral hepatitis is a major health problem worldwide. HBV and HCV are becoming endemic for the entire world. HBV and HCV cause coinfection due to the same transmission mode [4, 7]. Investigating the coinfection HBV and HCV is crucial as it may accelerate the course of the liver damage, increase the risk of hepatocellular carcinoma, and affect therapeutic response to antiviral molecules [7–10].

In the present study, a total of 845 hospitalized and nonhospitalized suspected viral hepatitis patients were screened for the serodetection of HBV and HCV infection. It was observed that anti-HCV positivity was much higher (23.5%) compared to HBsAg (5.3%). Another study from Rawalpindi reported HBsAg prevalence (2.7%) and HCV positivity (10.4%). These contradictions may exist due to the study on general population and also immunochromatographic techniques (ICT) used for anti-HCV detection by Gul et al. [13]; while in the present study we target hospitalized and nonhospitalized patients, ELISA was used for the detection HCV antibodies.

The observed coinfection rate of HBV and HCV was 1.30%, which is in line with a previous study conducted by Raja et al. which reported the overall coinfection rate of about 1.1% in viral hepatitis suspected patients referred by different hospitals and clinics [12]. The rate of HBV was more in male patients as compared with female patients which is already reported by Qureshi et al. [6]. This might be the different immune response evoked in both male and female patients. In this study age groups of 21–30 and 40–50 years showed highest frequency of HBV and HCV related hepatitis. Other studies also documented high prevalence in age group of 22–30 and age group above 40 [13–15]. The possible explanation might be the increased chances of infection in the mentioned age groups. It was observed that HBV was more prevalent in unmarried patients, as HBV can be transmitted through sexual contact [16], and thus these patients are free to indulge in more sexual activity. In case of HCV, married patients were more prone to infection which is in line with a previous study [17]. Furthermore, samples collected form General and Gastroenterology ward showed increased HCV and HBV infection rate.

## 4. Conclusions

It was concluded from the study that prevalence of HCV and HBV in Pakistan is at an increasing rate. Large-scale studies are needed to understand the epidemiology of HCV and HBV infections. The data of the current study will help in the effective prevention and control measures against HBV and HCV infection.

## Figures and Tables

**Table 1:** Incidence of HBV, HCV, and coinfection. *N* = 845.

Serological markers	Seroprevalence
	*n* (%)
HBsAg	45 (5.3)
Anti-HCV	199 (23.5)
Coinfection	11 (1.30)

Overall seropositivity	255 (30.1)

**Table 2:** Gender-wise seropositivity of hepatitis B, C, and coinfection. *n* = 845.

Serological markers	Gender		Total prevalence *n* (%)
	Male	Female	
	*n* (%)	*n* (%)	
HBsAg	29 (23.8)	16 (12)	45 (5.32%)
Anti-HCV	87 (71.3)	112 (84.2)	199 (23.55%)
Coinfection	06 (4.9)	05 (3.8)	11 (1.3%)

Total	122 (47.8)	133 (52.2)	255 (30.1%)

**Table 3:** Seropositivity of HBsAg, anti-HCV, and coinfection in different age groups.

Age groups	HBsAg	Anti-HCV	Coinfection	Total
	positive	positive	positive	+ (%)
1–9	01	03	00	04 (0.47%)
10–20	07	11	01	19 (2.24%)
21–30	18	53	03	74 (8.75%)^♠^
30–40	09	52	02	63 (7.45%)^♠^
41–50	08	40	03	51 (6.03%)^♠^
51–60	03	20	01	24 (2.84%)
61–70	00	15	01	16 (1.8%)
71–80	00	03	01	04 (0.47%)

Total	46	197	12	255 (30.1%)^♠^

^♠^Statistical analysis; univariate ANOVA. Significant (*P* < 0.05).

**Table 4:** Seropositivity of viral hepatitis in different marital status patients. *n* = 845.

Serological markers	Married	Unmarried	Total *n* (%)
HBsAg	21 (12)	25 (31.2)	46 (5.68%)^♠^
Anti-HCV	147 (84)	50 (62.5)	197 (23.3%)^♠^
Coinfection	07 (4)	05 (6.2)	12 (1.42%)

Total	175 (68.6)	80 (31.3)	255 (30.1%)^♠^

^♠^Statistical analysis; univariate ANOVA. Significant (*P* < 0.05).

**Table 5:** Seropositivity of hepatitis B, C, and coinfection in patients from different hospital wards and departments *n* = 845.

Departments	HBsAg	Anti-HCV	Coinfection	Total
	positive	positive	positive	+ (%)
General ward	15	70	02	87 (10.2%)^♠^
Gastroenterology	10	41	03	54 (6.39%)^♠^
General medicine	07	33	00	40 (4.73%)^♠^
Emergency	08	12	00	20 (2.36%)^♠^
Cardiology	02	08	00	10 (1.18%)
Nephrology	00	08	02 (18)	10 (1.18%)
Dental OPD	00	05	01 (9)	06 (0.71%)
Urology	00	05	00	05 (0.59%)
Ophthalmology	00	04	01 (9)	05 (0.59%)
Pulmonology	01	02	00	03 (0.35%)
Surgical ward	02	07	01 (9)	10 (1.18%)
Psychiatry	00	05	00	05 (0.59%)

Total	45	199	11	255 (30.1%)^♠^

^♠^Statistical analysis; univariate ANOVA, Duncan test. Significant (*P* < 0.05).
